# *Notes from the Field:* Vaccine Administration Errors Involving Recombinant Zoster Vaccine — United States, 2017–2018

**DOI:** 10.15585/mmwr.mm6720a4

**Published:** 2018-05-25

**Authors:** Tom T. Shimabukuro, Elaine R. Miller, Raymond A. Strikas, Beth F. Hibbs, Kathleen Dooling, Ravi Goud, Maria V. Cano

**Affiliations:** ^1^Division of Healthcare Quality Promotion, National Center for Emerging and Zoonotic Infectious Diseases, CDC; ^2^Immunization Services Division, National Center for Immunization and Respiratory Diseases, CDC; ^3^Division of Viral Diseases, National Center for Immunization and Respiratory Diseases, CDC; ^4^Division of Epidemiology, Center for Biologics Evaluation and Research, Food and Drug Administration, Silver Spring, Maryland.

Two vaccines for the prevention of herpes zoster (shingles) are licensed for use in the United States and recommended by the Advisory Committee on Immunization Practices (ACIP). Zoster vaccine live (ZVL; Zostavax, Merck), licensed in 2006,* is a live attenuated virus vaccine administered as a single subcutaneous (SQ) dose. Although the Food and Drug Administration (FDA) approved ZVL for adults aged ≥50 years, ACIP recommends ZVL for immunocompetent adults aged ≥60 years (*1*). Recombinant zoster vaccine (RZV; Shingrix, GlaxoSmithKline), licensed October 2017,^†^ is also approved by the FDA for adults aged ≥50 years and is recommended by ACIP for immunocompetent adults aged ≥50 years (*2*). RZV is administered as a 2-dose intramuscular (IM) series, with the second dose given anytime from 2 to 6 months after the first. RZV is preferentially recommended by ACIP over ZVL (*2*). Furthermore, ACIP recommends that persons previously vaccinated with ZVL receive the full 2-dose RZV series (*2*).

RZV and ZVL differ with regard to vaccine type, dose, and schedule; ACIP recommendation; route of administration; and storage requirements (Table). Prior experience indicates that administration errors are reported most frequently shortly after vaccine licensure and publication of recommendations, likely because of lack of vaccine provider familiarity with the new vaccine (*3*).

**TABLE:** Recommended storage, use, and administration of currently licensed herpes zoster (shingles) vaccines — United States, 2018

Characteristic	Brand name (manufacturer)	
	Shingrix (GSK)	Zostavax (Merck)
Vaccine type	Recombinant adjuvanted (RZV, licensed 2017)*	Live attenuated virus (ZVL, licensed 2006)^†^
Packaging	Supplied as 2 components: 1) single-dose vial of lyophilized varicella zoster virus glycoprotein E antigen and 2) a single-dose vial of AS01_B_ adjuvant suspension	Single-dose vial of lyophilized vaccine and a vial of sterile water diluent
Storage	Antigen and adjuvant should be stored refrigerated between 2°C and 8°C (36°F and 46°F); discard antigen or adjuvant components if frozen; discard reconstituted vaccine if frozen	Vaccine should be stored frozen between -50°C and -15°C (-58°F and +5°F),^§^ diluent should be stored separately at room temperature or refrigerated between 2° and 8°C (36°F and 46°F); do not freeze reconstituted vaccine
Reconstitution	Reconstitute the lyophilized varicella zoster virus glycoprotein E antigen component with the accompanying AS01_B_ adjuvant suspension component (single reconstituted dose is 0.5 mL)	Reconstitute lyophilized vaccine with the supplied diluent (single reconstituted dose is 0.65 mL)
Use	Administer immediately after reconstitution or refrigerate and use within 6 hours; discard reconstituted vaccine if not used within 6 hours	Reconstitute immediately upon removal of vaccine from the freezer and administer immediately after reconstitution; discard reconstituted vaccine if not used within 30 minutes
Route	Intramuscular (IM) injection	Subcutaneous (SQ) injection
Dose/Schedule	2 doses; second dose 2–6 months after the first dose	1 dose
Indication	Prevention of herpes zoster in adults aged ≥50 years	Prevention of herpes zoster in adults aged ≥50 years
ACIP recommendation	Immunocompetent adults aged ≥50 years, including those who previously received ZVL,^¶^ RZV is preferred over ZVL for the prevention of herpes zoster and related complications^¶^	Immunocompetent adults aged ≥60 years**

**Abbreviations:** ACIP = Advisory Committee on Immunization Practices, GSK = GlaxoSmithKline; RZV = recombinant zoster vaccine; ZVL = zoster vaccine live.

* Shingrix (zoster vaccine recombinant, adjuvanted) package insert. https://www.fda.gov/downloads/BiologicsBloodVaccines/Vaccines/ApprovedProducts/UCM581605.pdf.

^†^ Zostavax package insert. https://www.fda.gov/downloads/BiologicsBloodVaccines/Vaccines/ApprovedProducts/UCM132831.pdf.

^§^ ZVL (Zostavax) may be stored or transported at refrigerator temperature between 2°C to 8°C (36°F and 46°F) for up to 72 continuous hours before reconstitution; vaccine stored between 2°C to 8°C (36°F and 46°F) that is not used within 72 hours of removal from -15°C (+5°F) storage should be discarded.

^¶^ Recommendations of the Advisory Committee on Immunization Practices for use of herpes zoster vaccines. https://www.cdc.gov/mmwr/volumes/67/wr/mm6703a5.htm?s_cid = mm6703a5_w.

** Prevention of herpes zoster: recommendations of the Advisory Committee on Immunization Practices (ACIP). https://www.cdc.gov/mmwr/PDF/rr/rr5705.pdf.

During the first 4 months of RZV monitoring (October 20, 2017–February 20, 2018), the Vaccine Adverse Event Reporting System (VAERS) (*4*) received 155 reports involving RZV, 13 (8%) of which documented an administration error, including some reports documenting more than one error. Among these reports, nine involved RZV given by the SQ route rather than the IM route; injection site reactions (e.g., pain, erythema, and pruritus) were described in eight of these nine reports. One of the nine reports describing errors in the route of administration also described vaccination of a person aged 48 years (inappropriate age), and two described patients receiving the vaccine information statement for ZVL instead of RZV and not being instructed to return for the second RZV dose. The remaining four reports included 1) administration of RZV instead of the intended varicella (Varivax) vaccine to a person of unreported age, 2) administration of RZV after incorrect frozen storage, 3) administration of RZV to a person aged 39 years, and 4) administration of only the adjuvant component without reconstitution with the vaccine antigen. Vaccine administration errors occurred in a pharmacy (nine reports), a health care provider’s office (two), and unknown sites (two). CDC also received 13 public inquiries concerning RZV administration errors or questions asked to avoid errors. Topics included SQ administration (five), reconstitution (five), incorrect interval or schedule (two), and administration of previously frozen vaccine (one).

Although data from passive reporting to VAERS and inquiries submitted to CDC limit the ability to draw conclusions regarding the cause of the administration errors, early monitoring indicates that vaccine providers might confuse administration procedures and storage requirements of the older ZVL and the newer RZV. Failure to reconstitute the vaccine and administration of only one component of RZV also appears to be occurring, similar to errors observed for other vaccines that require mixing (*5*). Whereas RZV administered through the appropriate IM route is associated with high rates of local and systemic reactions (*2*), erroneous SQ injection can increase the likelihood of these episodes (*6*). In addition, some errors could potentially affect vaccine effectiveness. To prevent RZV administration errors, vaccine providers should be aware of prescribing information, storage requirements, preparation guidelines, and ACIP recommendations for herpes zoster vaccines (*1*,*2*).

## Footnotes

* Zostavax (zoster vaccine live) package insert. https://www.fda.gov/downloads/BiologicsBloodVaccines/Vaccines/ApprovedProducts/UCM132831.pdf.

† Shingrix (zoster vaccine recombinant, adjuvanted) package insert. https://www.fda.gov/downloads/BiologicsBloodVaccines/Vaccines/ApprovedProducts/UCM581605.pdf.
